# Pneumonia in myasthenia gravis: Microbial etiology and clinical management

**DOI:** 10.3389/fcimb.2022.1016728

**Published:** 2022-12-09

**Authors:** Manqiqige Su, Shan Jin, Kexin Jiao, Chong Yan, Jie Song, Jianying Xi, Chongbo Zhao, Zhirui Zhou, Jianming Zheng, Sushan Luo

**Affiliations:** ^1^ Huashan Rare Disease Center and Department of Neurology, Huashan Hospital, Fudan University, Shanghai, China; ^2^ National Center for Neurological Disorders, Huashan Hospital, Fudan Univeristy, Shanghai, China; ^3^ Department of Neurology, The First Affiliated Hospital of Anhui University of Chinese Medicine, Anhui, China; ^4^ Radiation Oncology Center, Huashan Hospital, Fudan University, Shanghai, China; ^5^ Department of Infectious Diseases, Huashan Hospital, Fudan University, Shanghai, China; ^6^ National Medical Center for Infectious Diseases, Huashan Hospital, Fudan Univeristy, Shanghai, China

**Keywords:** myasthenia gravis, pneumonia, mechanical ventilation, microbial etiology, antibiotic susceptibility

## Abstract

**Introduction:**

Patients with myasthenia gravis (MG) are prone to the development of pneumonia due to the long-term immunotherapies they receive and a tendency for aspiration. Pneumonia remains a risk factor for MG worsening and is the most prevalent cause of mortality in MG patients. Classification of the pathogens involved and exploration of the risk factors for mechanical ventilation (MV) could aid in improving clinical outcomes.

**Methods:**

Between January 2013 and October 2022, we performed an inpatient database review for MG patients with pneumonia concurrence in a tertiary research center specializing in neuromuscular disorders. The clinical and microbiological characteristics of 116 MG patients with pneumonia were retrospectively analyzed.

**Results:**

In our cohort, 90.32% (112/124) of organisms were bacteria and 42.86% (48/112) of pathogenic bacteria were carbapenem-resistant. A high abundance of Epstein–Barr virus (EBV) was detected using next-generation sequencing (NGS) in 12 patients, while cytomegalovirus (CMV) was detected in 8 patients. Non-fermentative Gram-negative bacilli were the most prevalent microorganisms, in which ampicillin, sulfamethoxazole-trimethoprim (SMZ-TMP), piperacillin, cefoperazone, ceftazidime, and cefepime may have an anti-infectious effect. Moreover, peripheral lymphocyte percentage [odds ratio (OR) 0.88, 95% CI 0.75–0.96, *p* = 0.02] and serum globulin (OR 1.16, 95% CI 1.02–1.35, *p* = 0.03) were significantly associated with the risk of MV demand.

**Discussion:**

Our identification of the microbial etiology of pneumonia in MG patients may provide future perspectives on accurate antibiotic options and enable early interventions when risk factors are present.

## Introduction

Myasthenia gravis (MG) is an autoimmune neuromuscular disorder associated with autoantibodies affecting neuromuscular junctions (NMJs) that leads to fluctuating weakness in extraocular, limb, bulbar, and even respiratory muscles (Punga et al., 2022). Approximately 85% of MG patients have autoantibodies against the acetylcholine receptor (AChR), and a small proportion of patients have antibodies against muscle-specific tyrosine kinase (MuSK) (Lazaridis and Tzartos, 2020). Anti-low-density lipoprotein receptor-related protein 4 (LRP4) antibodies were detected in approximately 2%–46% of MG patients who were negative for both AChR and MuSK antibodies (Higuchi et al., 2011). In triple-seronegative MG patients, 13.4% were titin antibody positive (Stergiou et al., 2016). Among all causes of MG mortality, pneumonia is the most prevalent cause, ranging from 4% to 21.7% across different ethnicities (Owe et al., 2006; Chen et al., 2020; Westerberg and Punga, 2020). In addition, pneumonia is a well-recognized risk factor for MG worsening and is associated with an increase in intensive care unit (ICU) admission rates, length of hospital stays, mortality, and poor outcomes (Thomas et al., 1997; Bershad et al., 2008; Tiamkao et al., 2014; Gummi et al., 2019). Many studies have been published regarding the effects of COVID-19 and relevant vaccines on MG in recent years (Jakubikova et al., 2021; Lupica et al., 2022).

Pneumonia is most common in all concurrences of infection among MG patients, ranging from 16% to 41.18% (Prior et al., 2018; Sipila et al., 2019; Kassardjian et al., 2020). The high susceptibility of pneumonia in MG patients is mainly related to fluctuated muscle weakness. About 70% of generalized MG (GMG) patients have bulbar muscle weakness and swallowing dysfunction that is significantly associated with aspiration (Hehir and Silvestri, 2018; Kumai et al., 2019). Also, the proportion of respiratory muscle involvement increases from 1% to 80% if the disease progresses into a more advanced stage (Engel, 1994). Longitudinal studies revealed a significant decrease in maximum voluntary ventilation capacity to 35% to 62% of the expected value (Heliopoulos et al., 2003). Moreover, MG worsening impairs the oropharyngeal tract movement and reduces airway clearance (Galassi and Marchioni, 2021). Approximately 15%–20% of GMG patients develop a life-threatening condition with respiratory failure, named myasthenic crisis (MC), and require invasive or non-invasive mechanical ventilation (MV) (Thomas et al., 1997). Of these, MC patients with endotracheal intubation may develop ventilator-associated pneumonia (VAP), which approximately accounts for 50% of hospital-acquired (nosocomial) pneumonia (HAP) (Zaragoza et al., 2020).

In addition to the above risk factors, an immunocompromised status also contributes to a high incidence of pneumonia in MG cohorts. The mainstay in current standard-of-care therapies for MG includes corticosteroids and immunosuppressants (Verschuuren et al., 2022). Consequently, long-term immunotherapies increase the probability of infection and can have potentially worse outcomes.

Although predisposition to pneumonia, clinical features and the prognostic factors of pneumonia in MG patients have not yet been well described. There was a lack of information about the microbiology of causative pathogens and antibiotics treatments supported by etiological evidence. Aiming to optimize the initial antibiotic therapies and improve the outcome of MG patients with pneumonia, this study retrospectively reviewed the causative pathogens for pneumonia and the drug resistance results, and explored the predictors for the unfavorable outcome of MV.

## Methods

### Study population

This is a retrospective cohort study conducted at a tertiary MG diagnostic center. We retrieved information on MG patients with the concurrence of pneumonia from the inpatient database at Huashan Hospital, Fudan University from January 2013 through October 2022. The diagnosis of MG was based on the criteria after excluding other MG mimicking diseases (Narayanaswami et al., 2021). Patients with a short hospital stay (<3 days) were excluded from our analysis due to incomplete records. Late-onset MG (LOMG) was defined as the disease onset after the age of 50 years and had no concurrence of a thymoma.

Pneumonia was diagnosed according to the American Thoracic Society/Infectious Diseases Society of America (ATS/IDSA) guidelines (Mandell et al., 2007; Metlay and Waterer, 2020). Community-acquired pneumonia (CAP) was defined as an acute infection of pulmonary parenchyma acquired in the community and VAP was defined as pneumonia that arose more than 48 h after patients have been intubated. Hospital-acquired pneumonia (HAP) indicated that pneumonia not associated with MV, which occurs at least 48 h after admission, and that it was not incubating at the time of admission (Kalil et al., 2016). There was no COVID-19 patient in this cohort.

### Clinical and laboratory variable collection

Clinical and laboratory data of MG patients were retrospectively reviewed. Baseline laboratory results included in the analysis were limited to those sampled within 24 h after admission. The CURB-65 score and SIPF were used to assess the severity of pneumonia.

### Outcome measures

The primary outcome was defined as the MV demand during hospitalization in MG patients with pneumonia. To explore the risk factors for MV dependence, the clinical variables underwent further analysis including MG post-intervention status (PIS) before admission and laboratory results.

### Next-generation sequencing

The sputum or bronchoalveolar lavage fluid (BALF) cultures were collected and sent for next-generation sequencing (NGS) (BGI). Sputum/BALF (1.5–3 ml) and other samples from patients were collected according to standard procedures. Saponin was added to a 0.45-ml sample at a final concentration of 0.025%. Then, the sample was fully vortexed for 15 s and incubated for 5 min at 25°C; 75 μl was added for the dehosting process. The sample was fully vortexed for 15 s and incubated at 37°C for 10 min. Then, the sample was centrifuged at 18,000 *g* for 5 min and ~70–80 μl remained at the bottom after the removal of 450 μl of supernatant. PBS (800 μl) was added to the tube and fully vortexed. After centrifugation at 18,000 *g* for 5 min, 800 μl of supernatant was discarded and ~70–80 μl remained at the bottom. TE buffer (370 μl) was added to the tube, followed by shaking. Then, 7.2 μl of lysozyme was added for wall-breaking reaction. Two hundred fifty microliters of 0.5-mm glass beads were attached to a horizontal platform on a vortex mixer and agitated vigorously at 2,800–3,200 rpm for 30 min. The sample (0.3 ml) was separated into a new 1.5-ml microcentrifuge tube, and DNA was extracted using the TIANamp Micro DNA Kit (DP316, TIANGEN BIOTECH) according to the manufacturer’s recommendation.

Then, DNA libraries were constructed through DNA fragmentation, end repair, adapter ligation, and PCR amplification. Agilent 2100 was used for quality control of the DNA libraries. Quality qualified libraries were pooled, and DNA Nanoball (DNB) was made and sequenced by the BGISEQ-50/MGISEQ-2000 platform. High-quality sequencing data were generated by removing low-quality reads, followed by computational subtraction of human host sequences mapped to the human reference genome (hg19) using Burrows–Wheeler Alignment. The remaining data after the removal of low-complexity reads were classified by simultaneously aligning them to the Pathogens Metagenomics Database (PMDB), consisting of bacteria, fungi, viruses, and parasites. The classification reference databases were downloaded from NCBI (ftp://ftp.ncbi.nlm.nih.gov/genomes/).

The raw NGS data were submitted and the accession to these SRA data is PRJNA901187.

### Statistical analysis

The missing data of each parameter account for less than 5% and multiple interpolations were performed for missing values. Categorical variables were summarized as proportions and the differences between rates were tested by *χ* ^2^ or Fisher’s exact test, if appropriate. The Shapiro–Wilk normality test was applied for all continuous parameters to test normality. If the test values were below the level of significance (*p* = 0.05), the median and interquartile (IQR) range were used for the descriptive characteristics; otherwise, the mean and standard deviation (SD) were used to describe the data. To assess the continuous parameters between the two groups, the Mann–Whitney *U* test was used when the assumptions of the Student’s *t*-test were not met. The one-way ANOVA or the Kruskal–Wallis test compared three categorical groups, and the Bonferroni test was performed on each pair of groups.

The heatmap was performed by R according to the relative abundance of pathogens in the results of the PM-seq DNA test by the BGI group, and a *Z*-score normalization was performed on the normalized read counts across samples for each gene.

To identify the risk factors for an unfavorable outcome, univariate (statistical significance, *α* = 0.2) and multivariate (statistical significance, *α* = 0.05) logistic regression analysis with an odds ratio (OR) was performed. The assumption of linearity in the logit for the continuous variable was assessed by the Box–Tidwell test. Variance inflation factor (VIF) measured the degree of multicollinearity in a set of independent variables, and a VIF value that exceeded 5 indicated a problematic amount of multicollinearity. Variables showing statistical significance (*p* < 0.2) of univariate analysis were further included in multivariate logistic regression analysis (Mickey and Greenland, 1989; Lemeshow et al., 2013). Variables that did not reach the levels of statistical significance (*p* = 0.05) were eliminated and a new multivariate model was set. All data analysis and chart making were performed using R (v. 4.1.1).

## Results

### Clinical and laboratory features of MG patients with pneumonia during hospitalization

**Figure 1:**
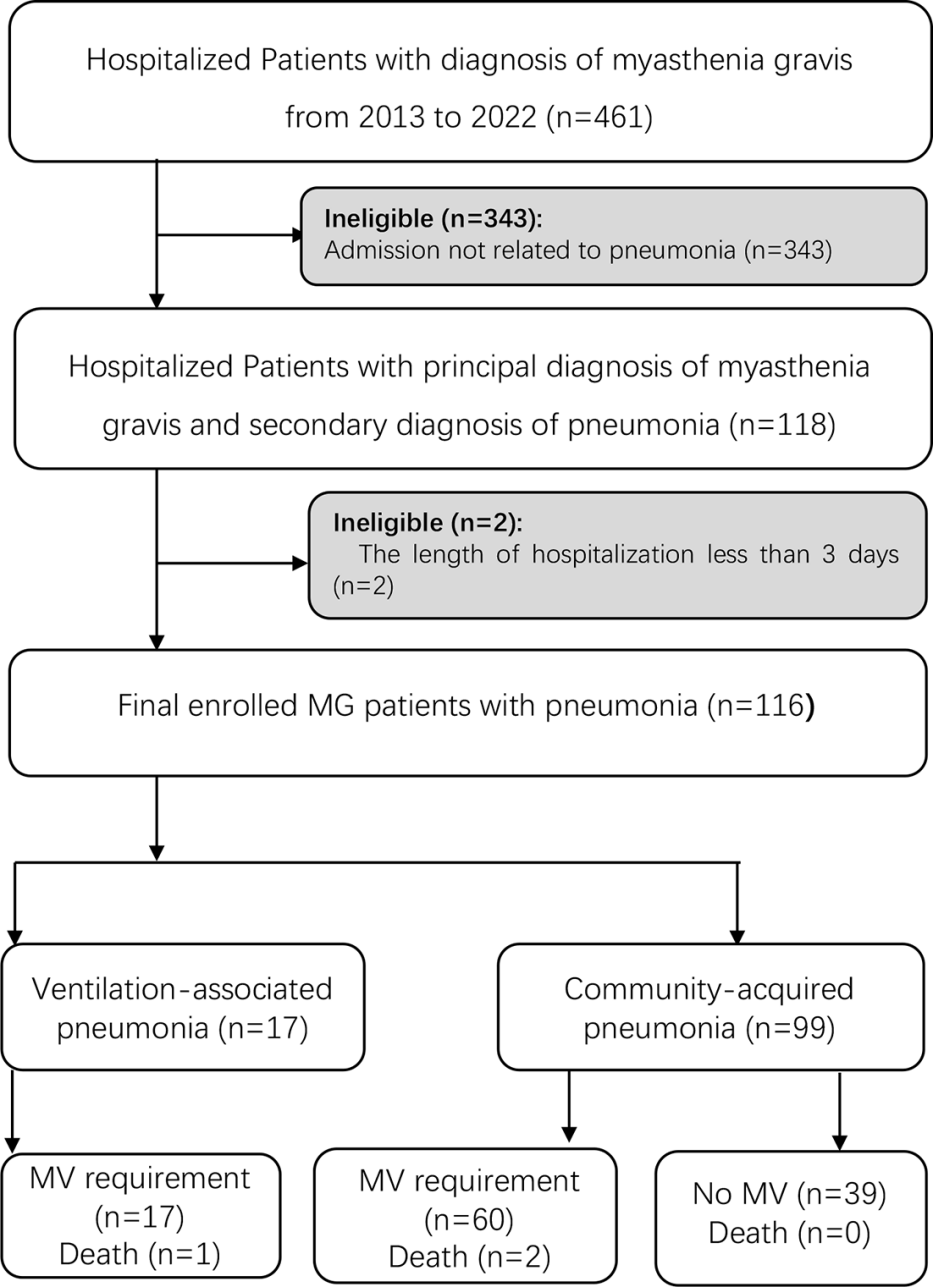
Flow diagram of patients included in the study. A total of 88 MG patients diagnosed with pneumonia were enrolled in the study. MG, myasthenia gravis; MV, mechanical ventilation.

We recruited a total of 461 patients who met the diagnostic criteria of MG in the inpatient database, including 146 patients with MC and 315 patients with non-MC. Among 146 patients with MC, 86 patients required MV without pneumonia. A total of 116 patients had pneumonia (116/461, 25.16%), among which 85.34% (99/116) had CAP and presented dyspnea before or shortly after admission. Ten patients had two episodes of pneumonia within 1 year before hospitalization, while others did not; 14.66% (17/116) met the diagnosis of VAP in our cohort. A total of 76 patients required MV treatment during hospitalization (**Figure 1**).

Three patients who demanded MV support eventually died with an average hospital length of 12.83 days. The in-hospital mortality is 2.59% (3/116); 76.72% (89/116) of enrolled patients had pneumonia-associated hospitalization for the first time, and the general length of hospital stay was 30.54 days. Furthermore, the average age at admission was 52.91 ± 15.04 years, and 54.31% (63/116) of patients were female, 15.51% (18/116) had the comorbidity of hypertension, and 7.76% (9/116) had diabetes upon admission.

Among 83 patients who had antibody testing, 81.93% (68/83) had autoantibodies against AChR, 8.43% (7/83) against MuSK, 26.51% (22/83) against titin, and 0 against LRP-4, and 9.64% (8/83) were seronegative. The age at onset was 48.68 ± 15.4 years and the average duration was 4.26 years. LOMG comprised 50% (58/116) of participants; 58.62% of patients (68/116) had thymoma concurrence, and 52.59% (61/116) received thymectomy with a mean duration of 3.7 years. At admission, 62.07% (72/116) were on corticosteroids and 31.9% (37/116) were on oral immunosuppressant therapies. Tacrolimus was the most prevalent immunosuppressant, which accounted for 21.55% (25/116). Prednisone was widely used as an oral corticosteroid, and the average dose was 29.21 ± 14.6 mg.

To assess the physiological state related to pneumonia when hospitalized, some pneumonia scores and laboratory results relevant to the pneumonia were retrospectively reviewed. CURB-65 was 0.55 on average, ranging from 0 to 3, and the mean SIPF score was 0.95. The white blood cell counts [(10.73 ± 5.1) ×10^9^ cells/L] slightly increased. The proportion and the absolute number of neutrophils rose to 80.49% and 10.09×10^9^ cells/L, respectively, while the average proportion of lymphocytes declined to 12.09%. The level of albumin/globulin ratio (A/G ratio, 1.11 ± 0.52) and hematocrit (HCT, 38.07%) decreased relatively. Arterial blood gas analysis revealed acidosis in 16 patients, alkalosis in 30 patients, and hypoxemia in 8 patients with PaO_2_ less than 8 kPa.

### Microbial etiology and antibiotic resistance associated with pneumonia

In the sputum or BALF cultures, 72 patients’ cultures were positive with a total number of 124 detected organisms. Among these patients, 48.61% (35/72) had mixed infections. Non-fermentative Gram-negative bacilli were the most common bacteria and accounted for 54.46% (61/112), including *Pseudomonas aeruginosa* (28.57%, 32/112), *Acinetobacter baumannii* (16.96%, 19/112), and *Stenotrophomonas maltophilia* (8.93%, 10/112). Given that different immunosuppressive treatments before hospitalization may have an impact on the microbiology of pneumonia, the prevalence of *Klebsiella pneumoniae* was 4.17 times higher in patients with corticosteroid treatment than those who did not (OR 4.17, 95% CI 1.22–19.55, *p* = 0.038).

**Figure 2:**
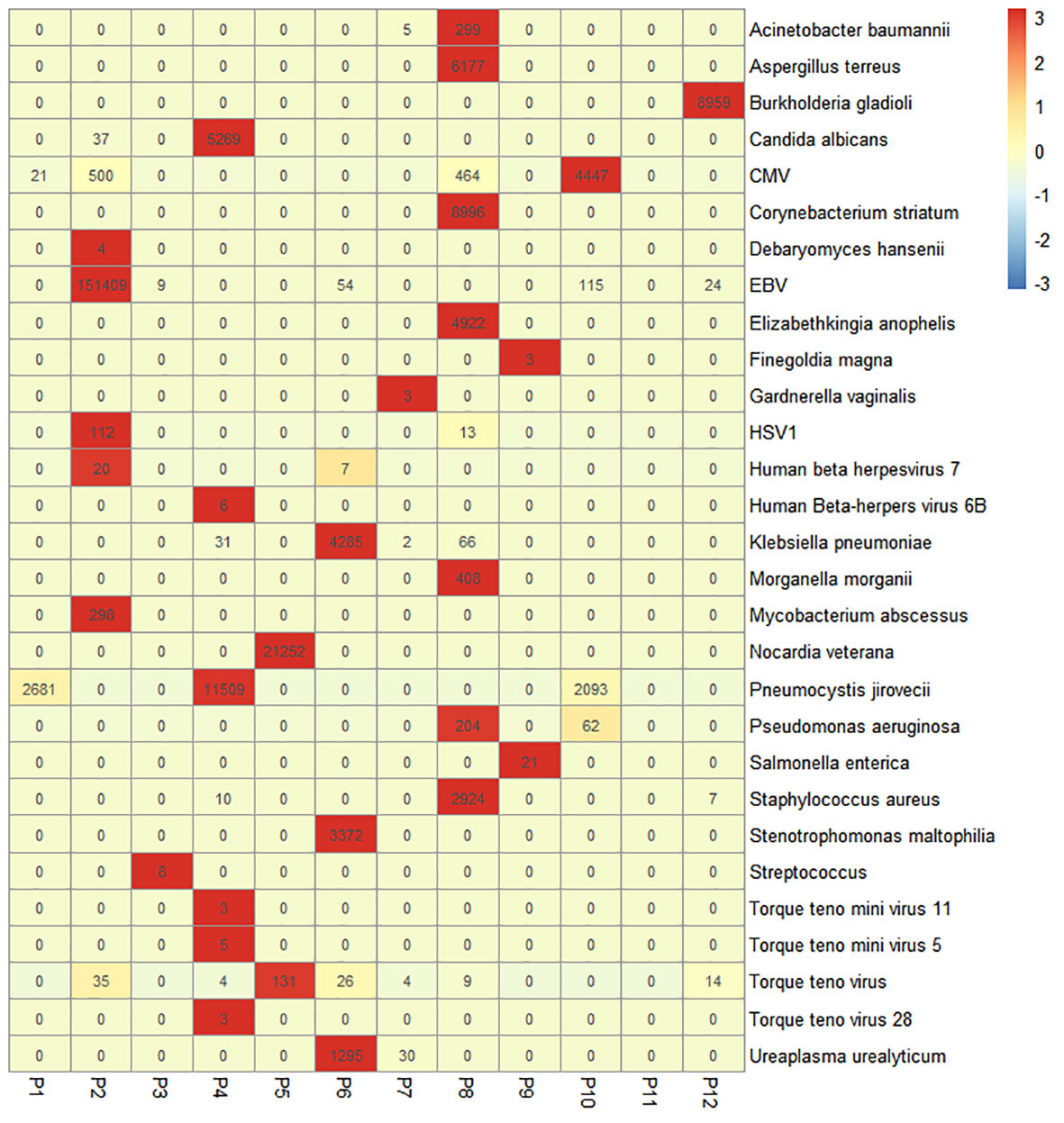
Heatmap of the pathogenic microorganism DNA/RNA high-throughput genetic sequencing (PMseq) results in 12 patients. A total of 29 pathogens were identified from 11 patients (91.67%, 11/12) including 15 bacteria, 4 fungi, and 9 viruses with different abundance. CMV, cytomegalovirus; EBV, Epstein–Barr virus; HSV1, herpes simplex virus 1.

For 12 patients whose sputum/BALF samples were analyzed by the NGS technique, we identified a total of 29 pathogens from 11 patients. In particular, Epstein–Barr virus (EBV) and cytomegalovirus (CMV) were frequently identified by NGS (**Figure 2**). The diagnostic yield of NGS was 91.67%, which was higher than that of traditional culture (7/12, 58.33%). There was a significant statistical difference in the detection sensitivity between NGS and traditional methods (*p* < 0.001).

**Figure 3:**
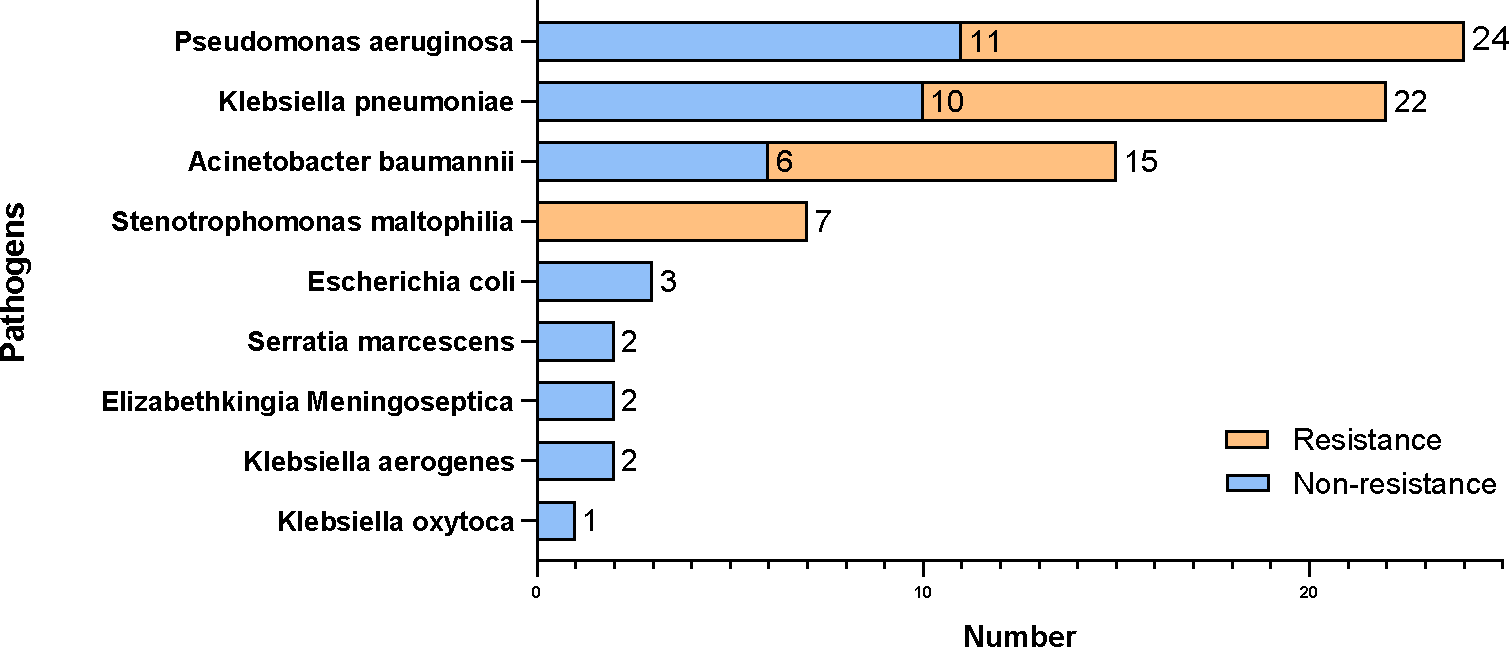
Results of microorganism culture and carbapenem resistance profile.

**Figure 4:**
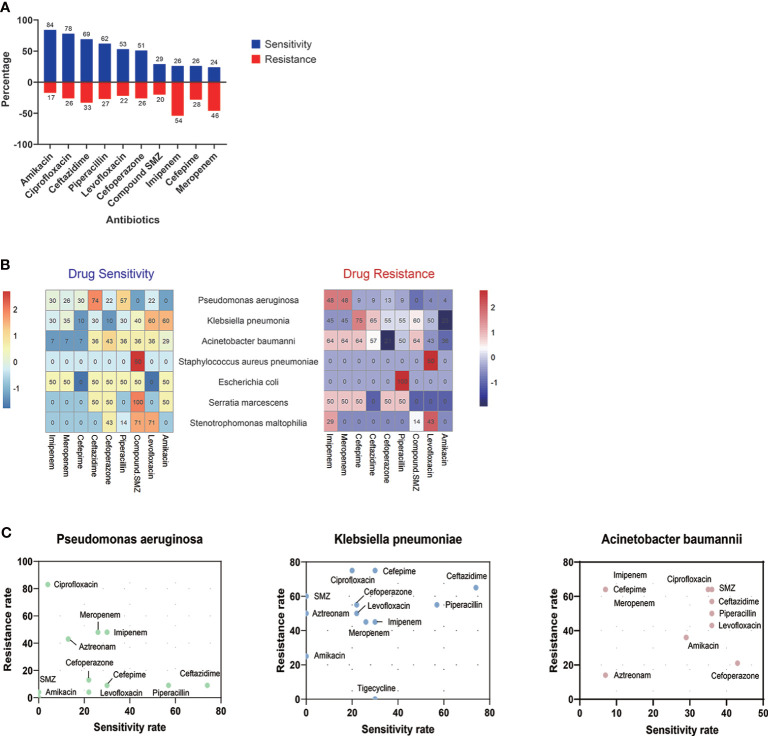
Drug resistance and sensitivity results of 112 bacteria. Amikacin (84/116, 72.41%) and ciprofloxacin (78/116, 67.24%) had high-sensitivity rates among 10 antibiotics, while bacteria detected were highly resistant to imipenem (54/116, 48.27%) and meropenem (46/116, 39.66%) **(A)**. The results of antibiotic resistance and sensitivity in different drugs **(B)** and three main pathogens **(C)** are presented. SMZ, sulfamethoxazole.

About 90.32% (112/124) of organisms were bacteria, among which 42.86% (48/112) of pathogenic bacteria were carbapenem-resistant (**Figure 3**). According to the drug sensitivity and resistance profile of 112 bacteria, carbapenem drugs, such as imipenem (54/112, 48.21%) and meropenem (46/112, 41.07%), had the highest resistance rate in the drug resistance tests. The antimicrobial susceptibility test highlighted amikacin, to which the microorganisms were most susceptible (84/112, 75%) (**Figure 4**). A total of 35 strains of bacteria were identified as MDRO. In particular, 10 strains were carbapenem-resistant *K. pneumoniae* (CRKP), among which 8 strains were in the MV group. In addition, 13 strains were carbapenem-resistant *P. aeruginosa* (CRPA), 9 strains were carbapenem-resistant *A. baumannii* (CRAB), and 3 strains were methicillin-resistant *Staphylococcus aureus* (MRSA).

### Clinical management for pneumonia in the MG cohort

Cefoperazone sodium/sulbactam sodium is the common initial antibiotic for our patients (62.07%, 72/116), and piperacillin tazobactam was used in 11.21% (13/116) of patients as the initial antibiotic therapy. In all antimicrobial regimens during hospitalization, β-lactam antibiotics were the most common drugs, which accounted for 82.76% (96/116), and it was highly used as monotherapy (70.83%, 68/96). Combination antibiotic therapies were used to treat 27 patients with the most common combination of β-lactam plus sulfonamides. Compared with the non-MDRO group, there was a significantly higher proportion of combined antibiotics in the MDRO group (*p* < 0.01).

As for ventilatory support, 65.51% of patients (76/116) were MV-dependent due to respiratory failure, namely, 41 invasive and 35 non-invasive MV. The average duration of MV during hospitalization was 25.39 (1–133) days. Of these, the length of MV demand in the invasive cohort was significantly longer than that in the non-invasive cohort (33.23 vs. 17.12 days, *p* < 0.01).

### Predictive factors for an unfavorable outcome in MG patients with pneumonia

MV demand was defined as an unfavorable in-hospital outcome. Patients were divided into three groups: VAP, CAP with MV, and CAP without MV. Isolated microorganisms of pneumonia in the three groups were not different except the *K. pneumoniae*, which was more prevalent in the CAP patients with MV (*p* < 0.05). The difference between subgroups was statistically significant in nine parameters (**Table 1**). Larger proportion of patients with a treatment history of oral immunosuppressant within one year before admission (p<0.01). The average level of lymphocyte percentage, SO_2_, albumin, and A/G ratio was significantly higher in the non-MV group, which hinted a better clinical status (*p* < 0.05).

**Table 1:** Comparisons of clinical and laboratory characteristics in three subgroups.

Variable	VAP (*n* = 17)	CAP with MV (*n* = 60)	CAP without MV (*n* = 39)	*p*-value
**Clinical features at admission**				
Length of hospitalization stay (days), median (IQR)	27 (26)	27.25 (25)	13.5 (11.5)	**0.004**
Age at admission (years old), mean ± SD	53.53 ± 18.02	54.65 ± 13.64	49.95 ± 15.65	0.31
Disease duration of MG (years), median (IQR)	1 (5.33)	2 (4.93)	2 (5)	0.7
Thymectomy, *n* (%)	9 (52.94)	30 (50)	16 (41.03)	0.67
LOMG, *n* (%)	9 (52.94)	32 (53.3)	17 (43.59)	0.62
IS within 1 year before hospitalization, *n* (%)	3 (17.65)	13 (21.67)	16 (41.03)	0.08
CS within 1 year before hospitalization, *n* (%)	11 (64.71)	45 (75)	31 (79.49)	0.51
MGFA classification, *n* (%)				
IIa	2 (11.76)	7 (11.67)	7 (17.95)	0.76
IIb	1 (5.88)	5 (8.33)	7 (17.95)	0.34
IIIa	0	2 (3.33)	2 (5.13)	0.81
IIIb	0	12 (20)	11 (28.21)	**0.04**
IVa	0	0	1	0.53
IVb	2 (11.76)	34 (56.67)	11 (28.21)	**<0.001**
V	12 (70.59)	0	0	**<0.001**
**Baseline pneumonia score and laboratory test within 24 h**				
CURB-65, median (IQR)	0.5 (1)	0 (1)	0 (1)	0.66
SIPF, median (IQR)	1 (1)	1 (1)	1 (1)	0.14
WBC count (×10^9^/L), median (IQR)	10.61 (4.45)	9.8 (5.74)	8.13 (6.75)	0.22
Lymphocyte (%), median (IQR)	12.2 (11.3)	7.1 (7)	11.7 (17.85)	**<0.001**
Absolute count of lymphocyte (/μl), median (IQR)	1 (1.01)	0.66 (0.73)	1.19 (1.05)	0.26
Neutrophil (%), median (IQR)	79.5 (16.8)	86.05 (12.95)	79.2 (19.05)	**0.047**
Absolute count of neutrophil (/μl), median (IQR)	6.79 (4.92)	8.57 (5.12)	6.39 (8.15)	0.48
HCT (%), median (IQR)	34.6 (8.4)	36.7 (5.48)	37.8 (10.7)	0.28
SO_2_ (%), median (IQR)	98.9 (1.8)	96.9 (3.7)	97.75 (3.33)	**0.02**
PaCO_2_ (kPa), median (IQR)	6 (1.14)	5.665 (1.44)	5.75 (1.04)	0.9
PaO_2_ (kPa), median (IQR)	16.44 (8.73)	13.92 (7.7)	11.9 (5.77)	0.51
BE (mmol/L), median (IQR)	2.3 (4.1)	2.5 (5.68)	2.3 (3.55)	0.38
Albumin (g/L), mean ± SD	33.88 ± 5.85	33.68 ± 5.43	37 ± 4.63	**0.008**
Globulin (g/L), mean ± SD	37.24 ± 12.22	37.99 ± 10.66	32.52 ± 10.76	0.05
A/G ratio, median (IQR)	0.79 (0.42)	0.86 (0.4)	1.2 (0.53)	**0.03**

IQR, interquartile range; LOMG, late-onset myasthenia gravis; HCT, hematocrit; WBC, white blood cell; SO_2_, oxygen saturation; PaO_2_, partial pressure of oxygen; PaCO_2_, carbon dioxide; BE, base excess; NLR, neutrophil-to-lymphocyte ratio. P values less than 0.05 were highlighted in bold type.

To explore the risk factors that led to MV demand in the CAP group, we conducted a univariable logistic regression analysis and identified six variables with significance. The result of the Box–Tidwell and VIF test denied the presence of non-linearity between these variables and the logit and interactions between variables. Based on statistical as well as practical considerations, we fitted the multivariable model containing six independent covariates (**Table 2**). After the elimination of variables above the level of statistical significance in the first fit of a multivariate model, the second analysis finally revealed two important variables: lymphocyte percentage (OR 0.88, 95% CI 0.75–0.96, *p* = 0.02) and globulin (OR 1.16, 95% CI 1.02–1.35, *p* = 0.03).

**Table 2:** Univariate and multivariate logistic regression models for the severe course in the hospitalized MG patients with pneumonia.

Variables		Univariate analysis *			Multivariate analysis †		
		OR	95% CI	*p*-value	OR	95% CI	*p*-value
Thymectomy	Yes	1.43	0.64–3.28	0.38			
	No						
IS within 1 year before admission	Yes	0.39	0.16–0.96	**0.04**	0.59	0.2–1.71	0.33
	No						
CS within 1 year before admission	Yes	0.85	0.31–2.23	0.74			
	No						
Duration of MG		0.85	0.31–2.23	0.74			
WBC count		1.07	0.99–1.18	**0.11**	1.01	0.91–1.12	0.92
Lymphocyte (%)		0.91	0.86–0.95	**<0.001**	0.88	0.75–0.96	**0.02**
Absolute number of lymphocytes		0.85	0.55–1.14	0.34			
Neutrophil (%)		1.03	1.00–1.07	**0.04**	0.98	0.87–1.03	0.63
Absolute number of neutrophils		0.99	0.95–1.03	0.64			
HCT		0.98	0.94–1.01	0.34			
SO_2_		1.03	0.91–1.17	0.58			
PaCO_2_		0.93	0.7–1.24	0.62			
PaO_2_		0.1	0.96–1.04	0.82			
BE		1.04	0.97–1.14	0.38			
Albumin		0.88	0.8–0.96	**0.003**	0.87	0.75–0.13	0.05
Globulin		1.05	1.01–1.09	**0.02**	1.16	1.02–1.35	**0.03**
A/G ratio		0.27	0.1–0.66	**0.006**	14.15	0.56–444.21	0.11

OR, odds ratio; CI, confidence interval; CS, corticosteroid; IS, immunosuppressant; HCT, hematocrit; WBC, white blood cell; SO_2_, oxygen saturation; PaO_2_, partial pressure of oxygen; PaCO_2_, carbon dioxide; BE, base excess; NLR, neutrophil-to-lymphocyte ratio. P values less than 0.2 in univariate analysis and less than 0.05 in multivariate analysis were highlighted in bold type.

## Discussion

To the best of our knowledge, this is the first study to comprehensively analyze the microbial spectrum and drug resistance of pneumonia in MG patients, as well as the risk factors for MV demand in this cohort. With the identification of non-fermentative Gram-negative bacilli as the most prevalent organism and multiple organisms that were responsible for the majority of this cohort, we attempt to provide future perspectives for empirical antibiotic therapies.

In comparison to another autoimmune disorder, systemic lupus erythematosus (SLE) (Garcia-Guevara et al., 2018), the etiology spectrum in MG for concurrent pneumonia was different, as evidenced by the prevalent pathogen profile. *P. aeruginosa*, *K. pneumoniae*, and *A. baumannii* were commonly identified in MG patients, while *S. aureus*, *Pneumocystis* pneumonia, and *Aspergillus* were identified in SLE patients from Mexico. The NGS technique exhibited a higher detection sensitivity compared with the traditional cultures, especially for the detection of potential virus infection.

Pathogen spectrums in our cohort largely overlapped with HAP rather than CAP, which indicated the colonization transformation of the oropharynx with virulent organisms in MG patients under long-term immune-compromised treatments. It is worth noting that there is a significant increment of *K. pneumoniae* in CAP patients with MV (*p* = 0.03), whereas the drug resistance rate of *K. pneumoniae* also increased. The prevalence of *K. pneumoniae* was 4.17 times higher in patients who underwent corticosteroid treatment than those who did not undergo immunosuppressive therapies (OR 4.17, 95% CI 1.22–19.55, *p* = 0.038).

The clinical decision in selecting an appropriate antibiotic is essential for improving the clinical outcome. In our analysis, carbapenem drugs, including imipenem and meropenem, had the highest resistance rate in the drug resistance tests, while amikacin, ciprofloxacin, ceftazidime, and piperacillin had a higher rate of susceptibility. However, as macrolides and aminoglycosides impair neuromuscular transmission and may aggravate MG (Jones et al., 2011; Van Berkel et al., 2016), amikacin should be avoided in MG patients if there is another alternative. Moreover, fluoroquinolones may impair neuromuscular transmission, which should be cautiously used in MG patients (Sheikh et al., 2021). If there is no alternative therapeutic option, adverse drug reactions should be closely monitored. Given the insufficient adverse reaction reports of cephalosporins, sulfonamides, clindamycin, tetracyclines, polymyxin B, and nitrofurantoin, these drugs can be safely administered to MG patients. Briefly, according to the drug sensitivity test results of our study, ampicillin, sulfamethoxazole-trimethoprim (SMZ-TMP), piperacillin, cefoperazone, ceftazidime, and cefepime may have an excellent anti-infectious effect.

Antimicrobial agents that are active against MDRO, especially carbapenem-resistant Gram-negative bacteria, remain limited. Initial empirical anti-infection treatment should cover a variety of possible pathogens. According to the drug sensitivity results, enzyme inhibitors, tigecycline, ceftazidime-avibactam, polymyxin, and other drugs can be chosen.

To reduce the incidence of MV and intervene in the early stage, we also analyzed the relationship between clinical factors and the risk of MV demand. With OR less than 1, a higher lymphocyte percentage is associated with lower odds of MV demand, which may help identify patients with higher intendancy of MV at the beginning of hospitalization. This result indicates the importance of preadmission therapy status for MG patients. Regular measurements of immune systems including levels of immune system cells and immunoglobulin are necessary under the long-term immunosuppressive treatments, which helps early prevention of infections in MG patients. In addition, globulin is also a valuable predictor of disease severity and prognosis. However, as a higher level of globulin may be associated with pre-admission intravenous immunoglobulin use, whether the high levels of serum globulin are predictive of MV dependence requires further research in future prospective cohort studies.

The retrospective nature mainly limited the power of the study. The inherent shortage of conventional microbiological methods was also a limitation. Due to the time-consuming procedure for natural amplification and false detection of some culture-unfriendly microorganisms, the identification of pathogens always falls behind the early anti-infectious treatments. To satisfy the need for fast and precise infection diagnosis, new techniques with high efficiency and short turnaround time, such as NGS, should be applied more widely in future practice. We provided a precise diagnosis of disseminated *Talaromyces marneffei* infection assisted by NGS of multifarious specimens in an HIV-negative patient (Zhu et al., 2018). We used NGS to monitor disease progression and therapeutic efficacy in central nervous system infection (Ai et al., 2018). The NGS can even detect whether there is a carbapenem resistance gene in samples, enabling us to know the drug resistance of pathogens earlier. Thus, we used high-throughput sequencing technologies to track carbapenem-producing *K. pneumoniae* outbreak in an intensive care unit (Chen et al., 2019). Nanopore sequencing is another sequencing method, which is not used in the current study. However, we performed the first explorative study with nanopore sequencing in infectious endocarditis previously (Ai et al., 2020). In addition, we developed a rapid CRISPR-based assay for tuberculosis detection in various forms of direct clinical samples, which is also one of the pathogens causing pneumonia in MG patients (Ai et al., 2019). High-throughput sequencing technologies, including NGS, help overcome some limitations of the traditional culture, which includes a longer period of time for some special pathogens to report positive and non-comprehensive coverage of all the microorganisms. The application of high-throughput sequencing, which assists with rapid and accurate microbiological diagnosis, provides a new perspective on the clinical approach.

## Conclusion

In conclusion, we characterized the etiological spectrum and clinical management of pneumonia in MG patients. Subsequently, we analyzed the detective yield in the microbial spectrum between NGS and traditional cultures. Lower lymphocyte percentage and a higher level of globulin at admission were identified as risk factors for MV demand, which may lead to unfavorable clinical outcomes. Accurate antibiotic options and early identification of the risk factors are of paramount importance to improve the clinical outcome.

## Data availability statement

The raw data supporting the conclusions of this article will be made available by the authors, without undue reservation.

## Ethics statement

The studies involving human participants were reviewed and approved by Fudan University Huashan hospital. The ethics committee waived the requirement of written informed consent for participation.

## Author contributions

JZ and SL conceived the presented idea. MS, SJ, and KJ performed the computations and manuscript writing. CY, JS, and JX were involved in the interpretation of data. CZ and ZZ revised the manuscript. All authors contributed to the article and approved the submitted version.
