# Supplementary material for: Pneumonia in myasthenia gravis: Microbial etiology and clinical management
Source: Front Cell Infect Microbiol. 2022 Dec 9;12:1016728. doi: 10.3389/fcimb.2022.1016728 (PMC9780595; doi:10.3389/fcimb.2022.1016728)
Supplement: Supplementary file 1 [file Table_1.docx]

**Supplementary Materials**

Supplementary table 1. Difference of microbiological etiology among patients with different immunosuppressive treatments before hospitalization

|  | Pseudomonas aeruginosa | Klebsiella pneumoniae | Acinetobacter baumannii | Escherichia coli | Serratia marcescens | Elizabethkingia Meningoseptica | Klebsiella aerogenes | Klebsiella oxytoca |
| --- | --- | --- | --- | --- | --- | --- | --- | --- |
| Oral immunosuppressants (n=30) | 4 | 9 | 5 | 1 | 1 | 0 | 2 | 0 |
| Ciclosporin (n=1) | 0 | 0 | 0 | 0 | 0 | 0 | 0 | 0 |
| CTX (n=2) | 1 | 1 | 2 | 0 | 1 | 0 | 0 | 0 |
| Mycophenolate Mofetil (n=4) | 0 | 0 | 0 | 0 | 0 | 0 | 1 | 0 |
| Azathioprine （n=12） | 1 | 3 | 3 | 0 | 0 | 0 | 0 | 0 |
| Tacrolimus (n=25) | 4 | 7 | 4 | 1 | 1 | 0 | 2 | 0 |
| Rituxmab (n=4) | 0 | 0 | 0 | 0 | 0 | 0 | 0 | 0 |
| Steroids (n=84) | 21 | 29 * | 15 | 2 | 2 | 0 | 1 | 1 |
| IVIg (n=34) | 7 | 8 | 7 | 2 | 1 | 1 | 1 | 1 |
| PE (n=16) | 2 | 3 | 3 | 1 | 0 | 1 | 0 | 1 |

* Multivariate logistic regression: *p*<0.05
